# Duration of Pregnancy and Epoch of Conception

**Published:** 1890-07

**Authors:** 


### DURATION OF PREGNANCY AND EPOCH OF CONCEPTION.

Olshausen, Trans. Berlin Obst. and Gyn. Soc., 1888.

The new German civil code in its articles 1467 and 1572, contains important references to the *epoch of conception.* This term among jurists is synonymous with *duration of pregnancy* among physicians. For living children this period is limited to one hundred and eighty to three hundred days, both inclusive. A child born in matrimony is always to be considered legitimate unless the husband denies cohabitation during the period limited as above. By the term *living-born* child, forensic medicine usually understands one which has breathed before parturition was completed. Now, fœtuses of one hundred and eighty days are susceptible of respiration, but so are those of one hundred and seventy or one hundred and sixty days; hence the limit fixed by the code is too large. As to the term *vitality,* if this means capacity for continuous existence, the period of one hundred and eighty days is too short, for only those who have reached one hundred and eighty-nine to one hundred and ninety-six days of development have, as a rule, *vitality* in this sense. Again, the limit three hundred days is too short, for pregnancy has often continued three hundred and ten to three hundred and twenty days; and the author has had one which continued three hundred and twenty-four days. Hohl, Matthews, Duncan, and Kroche have fixed the extreme limit at three hundred and thirty-six, three hundred and twenty-five, and three hundred and thirty days respectively. With cows the normal duration of pregnancy is two hundred and eighty days, but it may extend to three hundred and twenty-one. In extra-uterine pregnancy, it has been demonstrated that the fœtus may remain alive for weeks after the termination of the normal period.

The following propositions are therefore suggested:

1. If respiration is to be considered evidence of a living child, the limits for that function should be fixed at one hundred and sixty to one hundred and sixty-five days.

2. If there is a question of vitality rather than mere ability to breathe, the limit should be fixed at one hundred and ninety-five days.

3. The extreme of three hundred days should be lengthened to three hundred and twenty to three hundred and twenty-five. For widows, the period of three hundred and twenty days may be established as a maximum period to establish legitimacy. A. F. C.—*Annals of Gynæcology and Pædiatry.*

